# Assessment of Nanoparticle-Mediated Tumor Oxygen Modulation by Photoacoustic Imaging

**DOI:** 10.3390/bios12050336

**Published:** 2022-05-13

**Authors:** Maharajan Sivasubramanian, Leu-Wei Lo

**Affiliations:** Department of Biomedical Engineering and Nanomedicine, National Health Research Institutes, Zhunan 350, Taiwan; 020609@nhri.edu.tw

**Keywords:** nanoparticles, photoacoustic imaging, oxygen carriers, oxygen generators, tumor oxygen saturation

## Abstract

Photoacoustic imaging (PAI) is an invaluable tool in biomedical imaging, as it provides anatomical and functional information in real time. Its ability to image at clinically relevant depths with high spatial resolution using endogenous tissues as contrast agents constitutes its major advantage. One of the most important applications of PAI is to quantify tissue oxygen saturation by measuring the differential absorption characteristics of oxy and deoxy Hb. Consequently, PAI can be utilized to monitor tumor-related hypoxia, which is a crucial factor in tumor microenvironments that has a strong influence on tumor invasiveness. Reactive oxygen species (ROS)-based therapies, such as photodynamic therapy, radiotherapy, and sonodynamic therapy, are oxygen-consuming, and tumor hypoxia is detrimental to their efficacy. Therefore, a persistent demand exists for agents that can supply oxygen to tumors for better ROS-based therapeutic outcomes. Among the various strategies, NP-mediated supplemental tumor oxygenation is especially encouraging due to its physio-chemical, tumor targeting, and theranostic properties. Here, we focus on NP-based tumor oxygenation, which includes NP as oxygen carriers and oxygen-generating strategies to alleviate hypoxia monitored by PAI. The information obtained from quantitative tumor oxygenation by PAI not only supports optimal therapeutic design but also serves as a highly effective tool to predict therapeutic outcomes.

## 1. Introduction

Photoacoustic imaging (PAI) is an emerging biomedical imaging technique that involves optical excitation of intrinsic contrast agents by near-infrared (NIR) light pulsed lasers, causing thermoelastic effects of photoacoustic signals. The transient expansion and contraction resultant from the absorption of NIR laser energy induce the generation of pressure waves that can be detected, received by ultrasound (US) transducers, and constructed into images. The co-operative use of intrinsic optical contrast agents and US detection endows them with both high spatial resolution and deep tissue penetration [1,2,3,4]. The three main advantages of PAI over traditional imaging techniques are: (1) the use of intrinsic contrast agents (hemoglobin, melanin, or lipids); (2) high spatial resolution and imaging at clinically relevant depths; and (3) ability to gather anatomical and functional information in real time. Due to these features, it is possible to obtain biologically relevant information, such as angiogenesis, tumor hypoxia, changes in tumor oxygen saturation (sO_2_), and total Hb concentration [5,6,7,8,9]. Several exogenous contrast agents for PAI are synthesized and currently being used [10,11,12,13,14,15].

Hypoxia is a prominent feature of solid tumors caused by uncontrollable proliferation and defective vasculature. This leads to an insufficient blood supply, producing a reduction in oxygen tension in tumor tissues (pO_2_ ≤ 2.5 mmHg), then promoting the invasion and metastasis of tumor cells [16,17,18]. In addition, hypoxia also increases tumor resistance to reactive oxygen species (ROS)-based therapies, such as photodynamic therapy (PDT, combination of light and photosensitizer (PS) to generate ROS), radiotherapy (RT, combination of radiosensitzer and X-ray to generate ROS), and sonodynamic therapy (SDT, combination of ultrasound and sonosensitizer to generate ROS). The therapeutic efficacy of PDT depends highly on oxygen, and oxygen consumption further aggravates hypoxia [19,20,21]. 

As a consequence, the development of strategies that can alleviate hypoxia by supplying oxygen to tumors for better therapeutic outcomes is crucial. For example, hyperbaric oxygen therapy (HBOT) involves breathing pure oxygen in a pressurized chamber [22,23,24]. A study demonstrated that HBOT improved tissue angiogenesis and tumor hypoxia, and it increased apoptosis [25]. Although encouraging, its broad application is constrained by certain disadvantages, such as the overproduction of ROS in healthy tissues, which causes hyperoxic seizures and barotrauma [26]. As an alternative, uses of nanomaterials for oxygen-releasing or oxygen-generation strategies are gaining significance. Due to their physio-chemical properties, on the one hand, nanomaterials can be tailor-made to accommodate oxygen-carrying materials and assist them to reach the targeted site of action and deliver oxygen [27,28,29,30]. For instance, perfluorocarbons (PFCs), due to chemical and biological inertness, as well as their ability to dissolve a large amount of oxygen, have been widely employed as artificial blood substitutes [31,32,33]. In a study, hollow PEG-Bi_2_Se_3_ NPs effectively accommodated oxygen-loaded PFC as an oxygen reservoir. When exposed to NIR light, a burst release of oxygen occurred and subsequently increased tumor oxygenation, which overcame hypoxia-associated radiotherapy resistance [34]. On the other hand, NPs could also increase oxygen saturation by the decomposition of endogenous hydrogen peroxide, which is overproduced by hypoxic tumors [35,36]. For example, albumin bound-MnO_2_ NPs increased tumor oxygenation by 45% by converting endogenous H_2_O_2_ in the tumor to oxygen. When combined with radiotherapy, it resulted in increased DNA double-strand breaks, which significantly inhibited tumor growth [37]. 

## 2. Biomedical Applications of PAI

**Figure 1:**
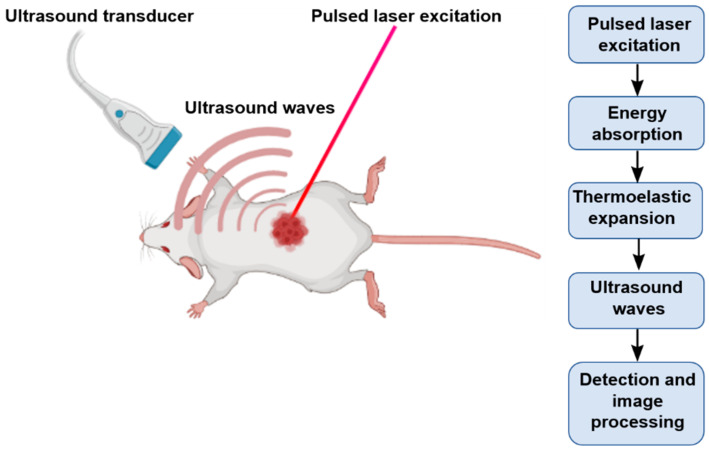
Schematic illustration of PAI.

PAI is a combination of optical and ultrasound imaging. When illuminated with a non-ionizing pulsed laser in the visible or near infrared wavelength, endogenous molecules absorb energy (Figure 1). Subsequently, the molecules in the ground state are elevated to an excited state, and they release energy in the form of light or heat when they relax back to the ground state. Through non-radiative relaxation, the absorbed photon is converted into heat. The induced heat mediates thermoelastic expansion, and thereby, pressure waves are generated. The pressure waves then propagate through tissues as ultrasound, which can be picked up and received by a US transducer to form an image [38,39]. Since sound waves are less scattered than photons, PAI can produce images with better optical contrast with high spatial resolution in deep tissues. In addition, since the image generated is based entirely on the optical absorption of endogenous molecules, PAI can deliver multi-contrast images of molecules based on their chemical composition. Owing to these features, PAI is successfully used to image endogenous molecules, such as oxy-Hb, deoxy-Hb, lipids, melanin, cytochrome, DNA/RNA, bilirubin, and water [40,41,42,43,44].

Breast cancer is the second-leading cause of cancer-related death in women, and the breast cancer mortality rate is continuing to increase [45,46]. Mammography is widely utilized as a main clinical diagnostic imaging technique for breast cancer [47,48,49]. However, high false-negative rates and decreased sensitivity with dense breast tissues remain challenging [50,51]. Breast tissues with high vascular density and increased oxygen saturation are potential markers. The vast potential of PAI, however, can easily overcome the limitations of mammography. An advanced SBH-PACT was developed that could image patients’ breast in 15 s and accurately detect tumors based on angiographic anatomy. The results obtained were verified with US-guided biopsy. Without the use of an external contrast agent, this breast imaging technique could monitor patients’ response to therapy [52]. 

Toi et al., precisely characterized the breast tumor microenvironment by visualizing oxygen saturation status using a new PAI system with a hemispherical detector array, which is otherwise not visible on standard contrast-enhanced magnetic resonance imaging (MRI). Interestingly, this system was also able to monitor anti-cancer treatment-driven changes in tumor vasculature, such as improved intratumoral blood perfusion and functional changes in intravascular Hb saturation of oxygen [53]. Dogra et al., performed ex vivo PAI analysis using endogenous biomolecules (for example: oxy-Hb, deoxy-Hb, lipids, and water) as chromophores to distinguish prostrate tumors from benign prostatic hyperplasia and normal prostrate. The authors discussed that the presence of deoxy-Hb as a main constituent in hypoxic prostrate tumors enabled them to detect malignant prostrate tissue [54]. Wang et al., developed a dual US/PAI system and tested its ability to quantify tissue oxygenation in vivo. The results showed that the established PAI system could readily image the vasculature in rat skin and quantify tissue oxygenation in an in vivo hypoxia model. Finally, in an orthotiopic pancreatic tumor mice model, real-time hypoxia dynamics was successfully assessed by measuring oxy-Hb and deoxy-Hb [55]. 

The potential of PAI for label-free, non-invasive quantification of tumor oxygenation as a biomarker of radiation response was tested in human papilloma virus-positive (HPV+) and -negative (HPV−) patient-derived xenograft (PDX) models of squamous cell carcinoma head and neck cancer. For this purpose, assessment of tumor oxygen saturation and Hb concentration before, during, and after fractionated radiation therapy (fRT) was performed. The results showed that HPV+ and HPV− xenografts exhibited a differential response to fRT. Modulation in oxygen saturation was observed within days after the initiation of fRT prior to palpable change in tumor volume [56]. In a PDX model of head and neck cancer, Seshadri et al., monitored the sO_2_% 24 h post RT and chemo-RT using combined PAI, MRI, and histopathology. An early increase in %sO_2_ was associated with significant tumor growth inhibition and, 24 h after RT, radiation-induced vascular damage was detected by PAI due to the loss of hemodynamic response to gustatory stimulation in murine salivary gland. This study established the utility of PAI in assessing both tumor and normal tissue changes in oxygen saturation and Hb concentration to radiation in head and neck cancers [57].

## 3. NPs as Oxygen Carriers or Generators

Hypoxia is a major factor in the tumor microenvironment, and it is characterized by depressed oxygen tension that encourages tumor cells to migrate, invade, and metastasize to distant organs. The partial oxygen pressure (pO_2_) of tumor hypoxia can be as low as 2.5 mm Hg compared to 40 mm Hg in normal cells, affecting cellular functions and causing improper functioning of organs. Hypoxia is either perfusion-related (temporary impairment in the delivery of blood) or diffusion-related (hampered movement of gas from blood capillaries to cancer cells) [58,59]. Furthermore, hypoxia severely diminishes the therapeutic performance of PDT, RT, and SDT, which strongly rely on oxygen to generate copious ROS to kill cancer cells [58]. The consumption of oxygen from tumors exacerbates hypoxia, raising the need for supplemental oxygen. In addition to several strategies, NP-mediated tumor reoxygenation is emerging and promising to alleviate hypoxia. This is because, by tailoring physiochemical properties, NPs can be made either to carry oxygen-philic materials or exhibit intrinsic enzyme-like properties to decompose endogenous hydrogen peroxides or self-decompose to generate oxygen in the tumor [30]. 

### 3.1. NPs with PFCs as Oxygen Carriers

PFCs are hydrocarbons, in which hydrogen atoms are completely or mostly replaced with fluorine atoms. The highly hydrophobic property of PFCs contributes to their biological inertness, which has the ability to dissolve oxygen. The interaction between PFCs and oxygen is not chemical but rather loosely bound to PFC macromers through van der Waals interaction [60,61]. Due to this feature, PFCs were used in MR and US imaging as a diagnostic imaging agent [62]. The dissolution equilibrium of oxygen in PFCs directly depends on oxygen partial pressure, which governs their loading and the release of oxygen at biological environments. Moreover, the oxygen-loading capacity is not influenced by temperature, ionic strength, surfactant, storage, etc. [63,64]. Attributable to the immiscibility of PFCs in water, PFCs for oxygen-carrying applications are usually emulsified with suitable surfactants as NPs for intravenous (i.v.) administration. For instance, Fluosol-DA emulsion in albumin was the first PFC system to be approved by the FDA. Due to its shortcomings, however, such as low oxygen transport capacity, premature oxygen release, short shelf-life, etc., the product has been withdrawn [65]. To overcome these limitations, by combining three different PFCs along with egg-yolk phospholipids, Oxygent was developed. This developed oxygen carrier displayed a diameter of 160–180 nm in diameter with reduced macrophage activation, and an ability to circulate in micro-capillaries to deliver oxygen. However, advanced clinical trials of the product in patients were terminated because of an increased risk of stroke and other adverse events [66]. 

### 3.2. NPs as Carriers for Oxygenphilic Materials

In order to improve oxygen level and enhance PDT efficacy, Cheng et al., developed lipid NPs loaded with a photosensitizer and perfluorohexane [67]. After i.v. injection into tumor-bearing mice, the oxy-PDT selectively accumulated in the tumor and maintained a high level of oxygen. When illuminated with laser light, the oxy-PDT generated abundant ROS in oxygen-enriched tumors and exhibited superior PDT effects compared to conventional PDT. It was also found that the lifetime of ^1^O_2_ was prolonged from 5 × 10^−6^  s in water to 5 × 10^−2^  s in perfluorohexane (PFH). This work demonstrated the effect of oxygen-rich tumors on PDT effects. Jiang et al., designed a hierarchical nanodroplet system (Au + perfluorooctylbromide (PFOB) + O_2_) to amplify DNA damage and inhibit the DNA repair mechanism in RT. PFOB, as an oxygen source, alleviated hypoxia and favored RT by elevated ROS production. Ultrasmall Au NPs, as radiosensitizers, confine X-ray energy to induce DNA damage. In vivo, the nanodroplets demonstrated multimodal imaging capability, through which real-time image-guided precision RT was realized. This strategy was not only able to reoxygenate the tumor but also inhibited DNA repair [68]. 

Zhao et al., integrated PS, oxygen reservoir, and tumor-penetrating peptide in a single nanoplatform (CNPs/IP). The results showed that CRGDK peptide with tumor-penetration property guided the tumor-specific accumulation and penetration of PS and PFOB into both tumor periphery and hypoxic regions. PFOB, as a reservoir, released oxygen in the tumor to alleviate hypoxia to enable enhanced PDT. In an MDA-MB-231 tumor model, CNP/IP demonstrated elevated PDT effects, as evidenced by a reduction in hypoxic regions in tumor tissues. The combined effects of improved intratumoral distribution of PS and adequate oxygen supply significantly improved PDT efficacy [69]. Similarly, Song et al., presented PEG-PFC nanodroplets decorated with tantalum oxide (TaOx) NPs via a simple emulsion method TaOx@PFC-PEG@O_2_. The high oxygen affinity of PFC allowed TaOx@PFC-PEG to be loaded with oxygen. In addition, TaOx NPs, as a radiosensitizer, was able to absorb X-ray and amplify DNA damage. In vivo, TaOx@PFC-PEG@O_2_ demonstrated the capability to increase oxygenation levels, which was followed by enhanced RT efficacy, by overcoming hypoxia-associated radio-resistance [70]. 

Zhou et al., developed a two-stage oxygen-delivery nanoplatform that consists of perfluorotributylamine (PFTA) as an oxygen reservoir loaded in albumin NPs. Among the various PFCs, the authors found that PFTA could increase RBCs infiltration and selected it as an oxygen carrier. In vivo, oxygen-saturated albumin NP selectively accumulated in tumor and increased oxygenation. Simultaneously, PFTA effectively increased RBCs infiltration and oxygen delivery by inhibiting platelet activation in tumor blood vessels. In vivo RT revealed a significant decrease in breast cancer tumor growth rate from 40% to 14% compared to control. In a colon cancer model, which is more hypoxic, the tumor growth rate decreased from 30% to 15% compared to control [71]. Zhang et al., constructed an “all-in-one theranostic amphiphile NPs featuring PEG-boron dipyrromethene amphiphile (PEG-F54-BODIPY)” to emulsify PFH into a theranostic nanoemulsion. The as-prepared nanoemulsion exhibited enhanced tumor accumulation, as evidenced by multimodal imaging ability and long tumor retention time. Due to these features, in a melanoma cancer xenograft model, the nanoemulsion demonstrated potential as an oxygen carrier and PS-quenching reliever to achieve highly efficient PDT [72].

**Figure 2:**
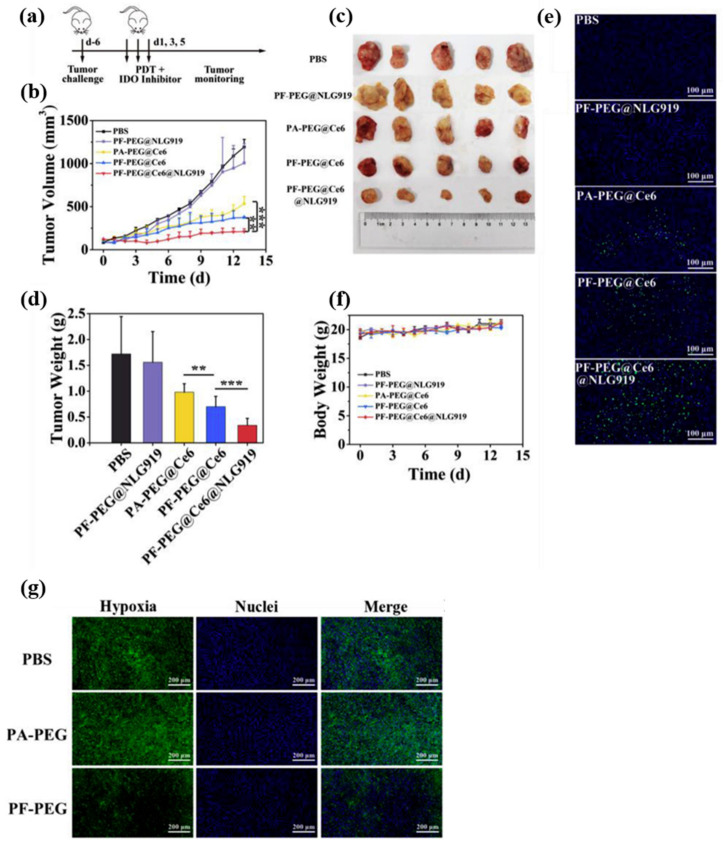
(**a**) The diagram of dosage regimen. (**b**) Inhibition of 4T1 tumor growth by PDT and IDO inhibitor. Tumor-bearing mice were intratumorally injected with oxygen-saturated PF-PEG@Ce6@NLG 919, and the tumor sites were illuminated (660 nm, 55 W/cm^2^). (**c**) Photograph of the tumors removed on day 14. (**d**) The weight of the tumor treated with different formulations on the last day. (**e**) TUNEL staining of tumor sections. The green light spots represent DNA damage tagged on FITC; the blue represents the nucleus labeled with DAPI. (**f**) The body weight of the mice. *p* values: ** *p* < 0.01, *** *p* < 0.001, one-way ANOVA, *n* = 5. (**g**) Immunofluorescence staining of tumor sections to detect whether the PF-PEG group could improve hypoxia in vivo. The nucleus and the anoxic region were stained with DAPI (blue) and SOSG (green), respectively. Reproduced with permission [74]. Copyright 2019, Elsevier B.V.

To eliminate hypoxia-induced drug resistance, Li et al., developed an EGFR-targeted liposome for the co-delivery of PFOB as an oxygen generator and erlotinib against hypoxic lung cancer. The targeted liposome selectively accumulated in EGFR-overexpressing cells and co-delivered oxygen and erlotinib, which induced apoptosis and down-regulated the expression of EGFR, p-EGFR, and HIF-1α. In lung tumor-bearing mice, the targeted liposome showed preferential accumulation in tumors, which was followed by the co-delivery of oxygen and erlotinib. Tumors were relieved of hypoxia-induced drug resistance, and a strong antitumor effect was consequently observed [73]. Xing et al., designed a multifunctional nanoplatform that employed fluorinated polymer NPs encapsulating Ce6 and an indoleamine 2,3-dioxygenase (IDO) inhibitor (NLG919). The fluorinated polymer NPs loaded with oxygen in advance released oxygen in the tumor, thereby diminishing hypoxia levels. The incorporation of IDO inhibitor along with PS greatly improved PDT efficacy by inhibiting the growth of primary and abscopal tumors via enhanced T cell infiltration (Figure 2) [74]. Yu et al., presented nano red blood cell (nnRBC) by replacing heme with perfluorodecalin (FDC). The developed formulation overcomes the autoxidative cytotoxicity and renal toxicity of heme, which demonstrated long-circulation, low immunogenicity, relieved hypoxia, and enhanced RT efficacy [75]. Chen et al., successfully developed an oxygen-generating SDT nanoplatform that encompasses FC-functionalized hollow mesoporous organosilica NPs for improving SDT performance. The well-defined mesoporous structure allowed high loading of a sonosensitizer (IR780), and the FC chain delivered oxygen in the tumor to mitigate hypoxia. In the presence of US, in vivo results showed enhanced tumor accumulation of NPs in hypoxic tumor, followed by accelerated oxygen release. In hypoxic PANC-1 pancreatic cancer, elevated ROS production resulted in highly efficient SDT by overcoming hypoxia-induced resistance [76].

### 3.3. NPs with Hb as Oxygen Carrier

The primary function of RBC is to transport oxygen from lungs to other tissues by binding to iron molecules containing the integral complex protein Hb. Hb is a globular protein that encompasses four polypeptide chains (two α and two β) folded onto itself. The three-dimensional folding pattern allows very efficient binding of a heme group. The tendency of oxygen to be either bound or released by Hb strongly depends on the partial pressure of oxygen [77,78,79]. Although the oxygen binding and releasing property of Hb is promising, free Hb possesses adverse side effects. For instance, after i.v. administration, patients suffered renal toxicity and cardiovascular complications. Cell-free Hb was also determined to have a very short circulation residence time because, when cell-free, Hb is unstable and dissociates into dimeric and monomer forms, and it is engaged by hepatobiliary and renal mechanisms leading to Hb-based toxicities in these organs. Moreover, free Hb is known to sequester nitric oxide, thus causing vasoconstriction and cardiovascular complications [80]. As an alternative, cross-linked Hb products were introduced, such as HemAssist and Optro, but these were associated with an increase in mortality rates. Then, several polymerized Hb products (Hemopure, PolyHeme, and HemoLink) were introduced by precisely controlling the polymer molecular weight. These products, however, showed a high risk of several complications in clinical trials [81]. 

Wang et al., established Hb-linked conjugated polymer NPs that do not require an external light source and mitigate hypoxia for efficient PDT. Hb acts as the catalyst for the luminol–H_2_O_2_ chemiluminescence system and supplies molecular oxygen. When luminol and H_2_O_2_ were extraneously added, PDT was initiated. A series of chemical reactions occurred, in which luminol, in the presence of H_2_O_2_ and Hb, radiated blue light while the polymeric NPs had an absorption in the range of 400–550 nm, which establishes a donor–acceptor for chemiluminescence energy transfer. As a consequence, Hb-bound NPs sensitize oxygen molecules by the absorption of luminol emission, producing ROS for enhanced PDT [82]. A multifunctional nanocomplex system was developed for oxygen-rich two-photon PDT that comprises two-photon absorbing molecules, PS and Hb, as an oxygen donor. PDT was initiated by the indirect activation of PS by two-photon laser through intraparticle fluorescence energy transfer, while Hb increased oxygen saturation in the tumor for improved PDT effect. Indeed, the nanocomplex developed in this study achieved improved PDT depth and mitigated hypoxia. Limited drug accumulation and the hypoxic tumor environment contribute to chemoresistance and lead to poor efficacy [83]. You et al., presented multifunctional liposomes for the synchronous delivery of oxygen and chemotherapeutic drug doxorubicin (DOX). Hb was embedded on the surface of the liposomes, while DOX was loaded inside of the liposomes. When i.v. administered, Hb enabled tumor-specific accumulation, which was followed by oxygen and DOX release. The oxygen released by liposomes alleviates hypoxia-driven chemoresistance that enabled DOX uptake in cancer cells. Due to the cooperative effects of tumor oxygenation and DOX release, liposomes exhibited stronger antitumor effects compared to controls [84]. Similarly, You et al., prepared liposomes for PDT against hypoxic tumors by encapsulating indocyanine green as PS and Hb as an oxygen donor. When i.v. administered, oxygen-carrying liposomes with PS demonstrated preferential accumulation in tumors. This led to an increase in tumor oxygenation levels, as evidenced by *T*_2_-weighted magnetic resonance imaging and immunostaining, and the level of hypoxia inducible factor-1α (HIF-1α) and vascular endothelial growth factor (VEGF) in the tumor was down-regulated. As a result, enhanced PDT efficacy against hypoxic tumor was observed compared to control groups after laser irradiation [85]. 

**Figure 3:**
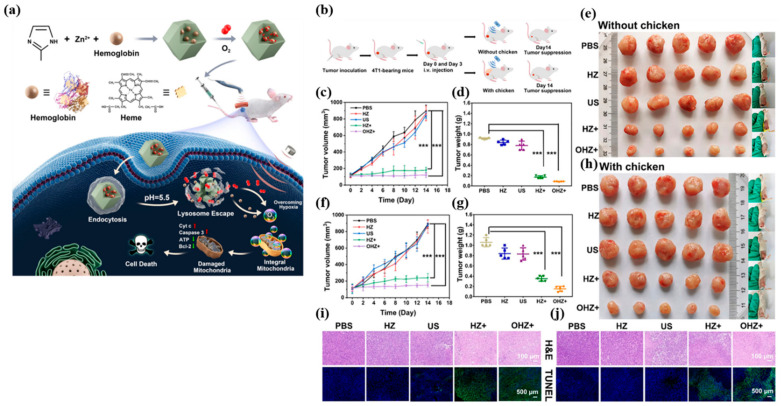
(**a**) Schematic illustration of the synthesis procedures and antitumor mechanism of OHZ NP. (**b**) Schematic illustration of OHZ NP for tumor treatment. (**c**) Relative tumor volume curves of mice treated with various treatments, (**d**) tumor weights of mice at the 14th day after the treatments, and (**e**) the photographs of mice and tumors in different groups at the end of treatments of the subcutaneous tumor treatment group. (**f**) Relative tumor volume curves of mice treated with various treatments, (**g**) tumor weights of mice at the 14th day after the treatments, and (**h**) the photographs of mice and tumors in different groups at the end of treatments of the deep-seated tumor treatment group mimicked by 2 cm chicken slice blocking. *p* values were calculated via ANOVA (*** *p* < 0.001). Fluorescence images of (**i**) subcutaneous tumor slices and (**j**) deep-seated tumor slices after being stained by H&E and TUNEL. Reproduced with permission [86]. Copyright 2021, American Chemical Society.

In order to maximize ROS generation efficacy by SDT, Hb was embedded in a zeolitic imidazolate framework (ZIF-8). In the as-prepared nanoplatform, Hb not only serves as an oxygen carrier but also exhibits the function of a sonosensitizer. In addition, the ZIF-8 shell in the NPs can be decomposed in the presence of low pH in tumor microenvironment to release oxygen, thereby alleviating hypoxia for enhanced SDT (Figure 3). In both subcutaneous and deep-seated tumors, NPs demonstrated improved SDT effects due to the cooperative effects of oxygen and SDT. The authors also found that this effective tumor inhibition was ascribed to activation of the mitochondrial apoptosis pathway [86]. As a strategy to diminish hypoxia and elevate the therapeutic effects of RT, Hu et al., presented Au-Hb NPs embedded with platelets. Here, platelets function as a tumor-targeting agent that could deliver radio-sensitizing Au NPs and oxygen-carrying Hb. In the presence of low-dose X-ray irradiation, Au NPs sensitized tumor cells to induce apoptosis through the formation of low-energy photons and secondary-charged particles with adequate O_2_ delivered by Hb. Although this nanoplatform achieved an antitumor effect with minimal side effects, clinical translation is not possible due to a high demand for platelets and translation-related challenges [87].

### 3.4. Oxygen Generation by Catalase (CAT) or CAT Mimicking NPs

CAT, an extraneous heme-containing enzyme that can rapidly decompose H_2_O_2_ into H_2_O and O_2_, is a highly suitable candidate for increasing tumor oxygenation. The expression levels of this enzyme in tumor, however, are lower than in normal tissues. Consequently, tumor-targeted delivery of CAT is essential to lower hypoxia levels, and the proteolytic degradation of CAT presents another challenge that needs to be addressed [88]. Several designed nanomaterials that can encapsulate or CAT have been reported [89]. The purpose of nanomaterials design is to preserve the enzyme activity of CAT during in vivo circulation and deliver them in tumors to increase oxygenation levels. A multifunctional CAT-loaded liposome with cisplatin (IV)-prodrug-conjugated phospholipid was developed by Liu et al., for chemo-radiotherapy [90]. The enzyme activity of CAT loaded inside of the liposome was preserved, and it decomposed endogenous H_2_O_2_ in tumor to mitigate hypoxia. When injected i.v. in a tumor mouse model, multifunctional liposome accumulated in the tumor and contributed to the highest level of DNA damage in cancer cells after X-ray radiation, and it further demonstrated an elevated therapeutic outcome in chemo-radiotherapy synergistically. Liu et al., presented an in situ free radical polymerization to modify CAT using meso-tetra(p-hydroxyphenyl) porphine (THPP), a photosensitizer, as the crosslinker and short-chained PEG as the drafting moiety. The obtained nanocapsules preserved the enzyme activity of CAT, as an oxygen generator can decompose H_2_O_2_ in tumor to relieve hypoxia for enhanced PDT. THHP not only functions as a PS but also acts as a chelating agent to label ^99m^Tc4+ for in vivo single-photon emission computed tomography (SPECT) imaging. When i.v. injected, nanocapsules passively accumulated in the tumor, as confirmed by SPECT imaging, and they generated oxygen by decomposing tumor-laden H_2_O_2_, which enabled remarkable PDT by destroying tumors [88]. 

Zhao et al., developed self-assembled NP for oxygen-boosted PDT. The nanosystem contains β cyclodextrin and CAT-conjugated hyaluronic acid loaded with adamantane- modified Ce6. The obtained nanosystem (HA-CAT@aCe6) maintained the enzyme activity and could accumulate in CD44 receptor-overexpressing cancer cells, thus generating adequate oxygen to abrogate hypoxia to elevate PDT. In vivo, HA-CAT@Ce6 actively accumulated in MDA-MB-231 tumor-bearing mice by the cooperative effects of oxygen and PDT, and significant inhibition in tumor volume was observed [91]. Zhang et al., constructed a biomimetic core–shell nanoplatform that consists of a pH-sensitive zeolitic imidazolate framework embedded with CAT and doxorubicin as the core and murine melanoma cell membrane coating as the shell. The core acts as a reservoir for drugs and an oxygen generator, whereas the shell provides tumor-targeting ability and elicits an immune response due to an abundance of antigens. In an in vivo tumor-bearing mouse model, core–shell NP reduced hypoxia levels, enhanced chemotherapeutic effects, and simultaneously down-regulated the expression of programmed death ligand 1 (PD-L1). When combined with immune checkpoints blockade therapy, the dual inhibition of the PD-1/PD-L1 axis elicited a strong immune response, prolonged tumor recurrence, and inhibited tumor metastasis [92]. 

Peng et al., developed a multifunctional CAT, i.e., DOX- and lysosome-targeted NIR PS MBDP-loaded liposome (FA-L@MD@CAT). It was demonstrated that FA-L@MD@CAT accumulated in tumor by both active and passive targeting mechanisms to increase tumor oxygenation for a remarkable chemo-combined PDT. In a tumor-bearing mouse model, FA-L@MD@CAT provided adequate oxygen for PDT and reversed immunosuppressive TME by modulating immune cytokines to elicit antitumor immunities, thus enhancing tumor inhibition in vivo [93]. Van Hest et al., designed a synergistic nanoplatform for MRI-guided tumor growth inhibition by boosting PDT efficacy. The nanoplatform consists of Ce6 conjugated glycol chitosan micelles loaded with CAT-stabilized MnO_2_ NP (CMGCC). GC provides a long in vivo half-life with a pH-stimulated charge switch for tumor accumulation, and MnO_2_ acts as an intracellular GSH scavenger to amplify ROS levels and also serves as a T_1_ contrast agent for MRI by releasing Mn^2+^. The systemic administration of CMGCC in a HeLa tumor demonstrated that it is a promising theranostic agent for PDT [94]. 

**Figure 4:**
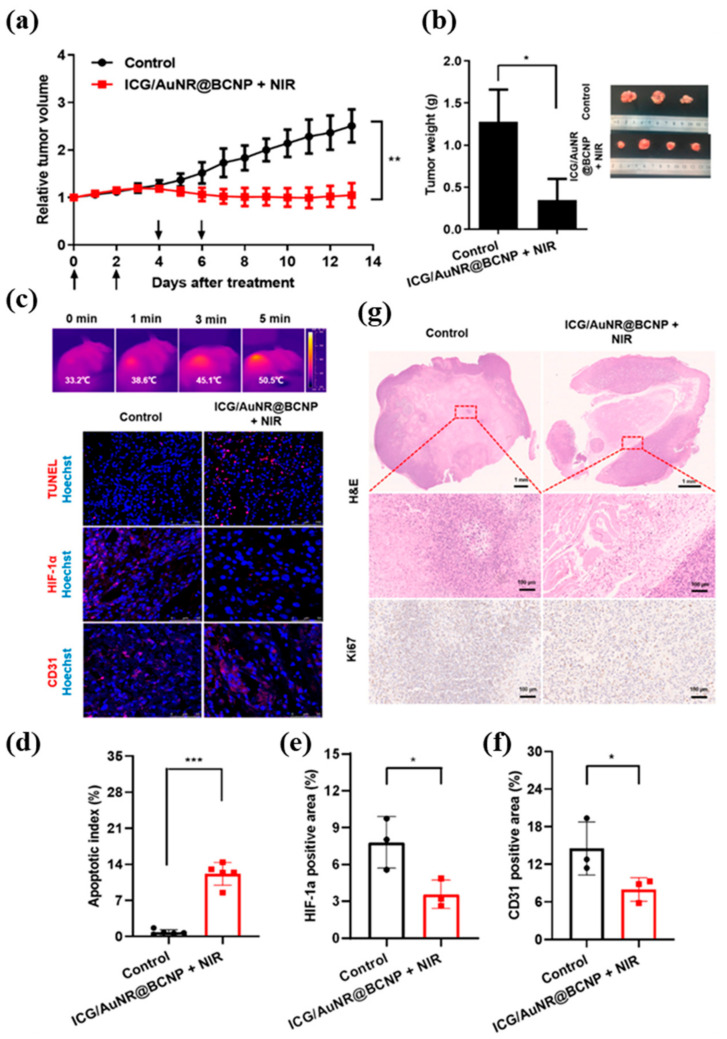
(**a**) Relative tumor growth curves recorded during treatment. The dosage of ICG/AuNR@BCNP in mice was equivalent to ICG 1.0 mg/kg and Au 0.76 mg/kg. (**b**) Weight and photograph of isolated tumor at the end of treatment. (**c**) Immunofluorescence staining of tumor sections with TUNEL, anti-HIF-1α, and anti-CD31 antibody. Scale bar: 50 or 100 μm. Corresponding semiquantitative analysis of (**d**) the apoptotic index and (**e**) HIF-1α and (**f**) CD31 positive areas (mean ± SD, *n* = 3–5, * *p* < 0.05, ** *p* < 0.01, *** *p* < 0.001). (**g**) H&E and ki67 staining of tumor sections. Scale bars: 1 mm for low magnification, 100 μm for high magnification. Reproduced with permission [95]. Copyright 2020, American Chemical Society.

Qi et al., combined multimodal imaging for diagnosis, guided surgery, and effective therapy to treat glioma (Figure 4). The developed CAT-integrated albumin theranostic nanoprobe (ICG/AuNR@BCNP) penetrated the blood–brain barrier and accumulated into glioma via albumin-binding protein-mediated transportation to perform theranostic functions. The combined fluorescence, PAI, and infrared thermal imaging were able to clearly differentiate brain tumors from surrounding tissues. The nanoprobe reduced hypoxia levels in glioma by generating adequate oxygen and induced elevated local hyperthermia. Through i.v. or i.t. in several animal models, guided by external multimodal imaging, nanoprobe inhibited glioma with improved apoptosis and antiangiogenic effects by remarkable phototherapy [95].

Manganese dioxide (MnO_2_) has a strong capacity to undergo CAT-like activity to decompose H_2_O_2_ to generate oxygen. In the presence of a mild acidic environment, MnO_2_ NP releases Mn^2+^ ions to promote the decomposition of H_2_O_2_ for oxygen generation and anticancer therapy. In addition to oxygen generation, Mn^2+^ ions could also be utilized as a T1 contrast agent for MRI [96]. Zhang et al., developed HA-MnO_2_ NP as an oxygen-modulating targeted MR imaging agent for glioma. NP was synthesized by toxicity-free simple mixing of sodium permanganate and HA solution, where HA serves as a reducing agent, a dispersing agent, and a CD44-targeting agent. After i.v. in a rat intracranial glioma model, HA-MnO_2_ NP exhibited sustained attenuation of tumor hypoxia by down-regulation of VEGF and HIF-1α expression. In the acidic tumor microenvironment, Mn^2+^ was released and demonstrated imaging sensitivity for detection with MRI for a prolonged period of up to 3 days. From these results, HA-MnO_2_ NPs has the capability for simultaneous targeted imaging, real-time monitoring, and tumor microenvironment modulation [97]. Guo et al., developed a self-assembled NP by mixing KMnO_4_ with HA solution followed by Ce6 loading for oxygen-assisted PDT to treat bladder cancer. The prepared HSA-MnO_2_-Ce6 NPs demonstrated oxygen generation with H_2_O_2_ that resulted in two-fold higher ROS production. In vivo, HSA-MnO_2_-Ce6 showed preferential accumulation in tumor, as confirmed by NIR and MR imaging with a ≈3.5-fold increase in oxygen levels. In an orthotopic bladder cancer mouse model, when combined with laser irradiation, it demonstrated remarkably improved therapeutic efficacy and significantly prolonged lifetime [98]. 

**Figure 5:**
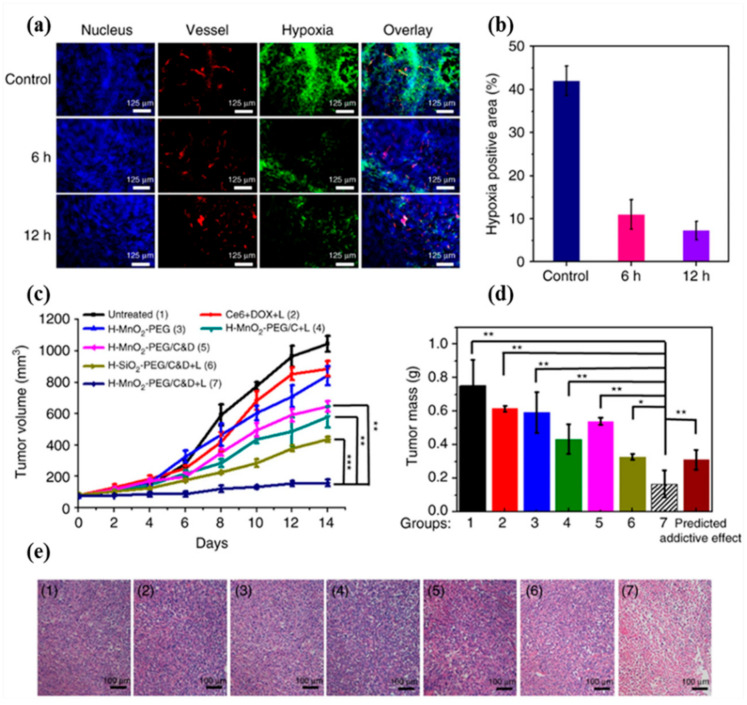
In vivo combined chemo-PDT treatment with H-MnO_2_-PEG/C&D. (**a**) Representative immunofluorescence images of 4T1 tumor slices collected from untreated control mice and mice 6 h and 12 h post i.v. injection with H-MnO_2_-PEG/C&D. The nuclei, blood vessels, and hypoxic areas were stained with DAPI (blue), anti-CD31 antibody (red), and anti-pimonidazole antibody (green), respectively (three mice per group). (**b**) Quantification of hypoxia areas in tumors at different time points post injection of our NP. (**c**) Tumor growth curves of different groups of mice after various treatments indicated. Error bars were based on standard errors of the mean (SEM) (six mice per group). (**d**) Average weight of tumors collected from mice at day 14 post initiation of various treatments. The predicted addictive effect was calculated by multiplying the tumor growth inhibition ratios of group 4 (PDT alone) and group 5 (chemotherapy alone). (**e**) H&E-stained tumor slices collected from mice post various treatments indicated. *p* values in (**c**,**d**) were calculated by Tukey’s post-test (*** *p*  <  0.001, ** *p*  <  0.01, or * *p*  <  0.05). Reproduced with permission [100]. Copyright 2017, Nature publishing group.

A composite core–shell NP was presented by Gang et al., for combined ROS-mediated PDT/CDT to treat breast cancer. The core–shell NP consists of indocyanine green loaded mesoporous silica as a core and MnO_2_ as a shell. MnO_2_ could not only generate oxygen in the presence of endogenous H_2_O_2_ but could also release Mn^2+^ ions to scavenge intracellular glutathione and generate ROS by Fenton-type reactions. In a breast cancer model in vivo, ICG-loaded nanozymes selectively accumulated in the tumor, and they inhibited tumor growth and metastasis by the cooperative effects of oxygen-boosted combined PDT/CDT [99]. A tumor microenvironment-responsive theranostic nanoplatform was developed by Liu et al., for combined PDT and chemotherapy favoring antitumor immunities. The nanoplatform is Ce6, DOX dual drug-loaded hollow mesoporous MnO_2_ shells (H-MnO_2_-PEG/C&D). The multifunctions of hollow mesoporous MnO_2_ shells are their ability to load drugs, release Mn^2+^ ions in acidic tumor microenvironment for tumor-specific MRI, and generate oxygen by decomposing endogenous H_2_O_2_ (Figure 5). Moreover, tumor oxygenation reversed the immune-suppressing mechanism by polarizing macrophages from M2 to M1 transition. Chemo/PDT combined with PD-L1 checkpoint blockade induced an abscopal effect, which not only inhibited primary tumors but also distant tumors without light exposure, likely through CTL migration as confirmed by subsequent T-cell depletion experiments [100]. 

Prussian blue (PB) NP has also been shown to perform CAT-like functions to decompose H_2_O_2_ to O_2_. Zhang et al., designed a mutt homologue 1 inhibitor and PS-loaded mesoporous silica-coated PB nanoplatform for oxygen-enhanced PDT. PB NP demonstrated an increase in tumor oxygenation, which could elevate ROS production to aggravate oxidative damage for cancer therapy by inhibiting the MTH1-mediated damage-repairing process [101]. In another study by Cai et al., porous hollow PB NP were embedded with glucose oxidase (GOx) and then modified with HA for active targeting to the tumor. PB NP of GOx actively accumulated in the tumor and decomposed H_2_O_2_ to O_2_ to reduce hypoxia levels and elevate glucose depletion for tumor starvation therapy [102].

## 4. PAI for the Assessment of NP-Mediated Tumor Oxygen Saturation 

Tumor hypoxia is a detrimental factor affecting the therapeutic outcomes of PDT, SDT and RT, because these therapeutic modalities strongly depend on oxygen tensions in tumor to induce ROS-mediated cancer cell death [58]. It is critical to identify the hypoxia status by quantifying tumor oxygenation. Specifically, it will not only assist clinicians to identify moderate-to-severely hypoxic tumors to plan treatment strategies, management and patient classification but also predict treatment efficacy in a very early stage, which will vastly improve patients’ quality of life. Invasive polarographic electrodes are the gold standard for the detection and characterization of tumors, but several factors have constrained their clinical application [103,104]. PAI offers non-invasive real-time monitoring and quantification of tumor oxygen saturation without the aid of exogenous contrast agents (Table 1) [8].

Hasan et al., used PAI and mapped changes in glioma tumor sO_2_ as a surrogate marker for predicting the probability of PDT success. PDT consumes oxygen in the tumor, which will cause a change in the tumor sO_2_. By measuring oxy-Hb and deoxy-Hb by PAI, a 3D atlas of tumor sO_2_ before, during, and after the PDT can be obtained. The authors found a ≈95% and ≈85% decrease in sO_2_ at 6 and 24 h post-PDT, and they predicted that tumors were responding to the treatment, as evidenced by no tumor recurrence observed up to one-month post-PDT. In contrast, no significant changes in sO_2_ were observed in the non-responding tumors. Information about the possibility of tumor regrowth in 24 h is crucial and presents the possibility of an early intervention. The tumor prediction sO_2_ map was validated with caliper measurements and photographs of the recurred tumor [99]. Similarly, Kolios et al., monitored DOX-loaded liposome treatment-induced changes in tumor vasculature using PAI. They found that quantitative PAI analysis at 30 min post-treatment correlated with a decrease in sO_2_ (22%) due to changes in tumor vasculature [106]. 

**Figure 6:**
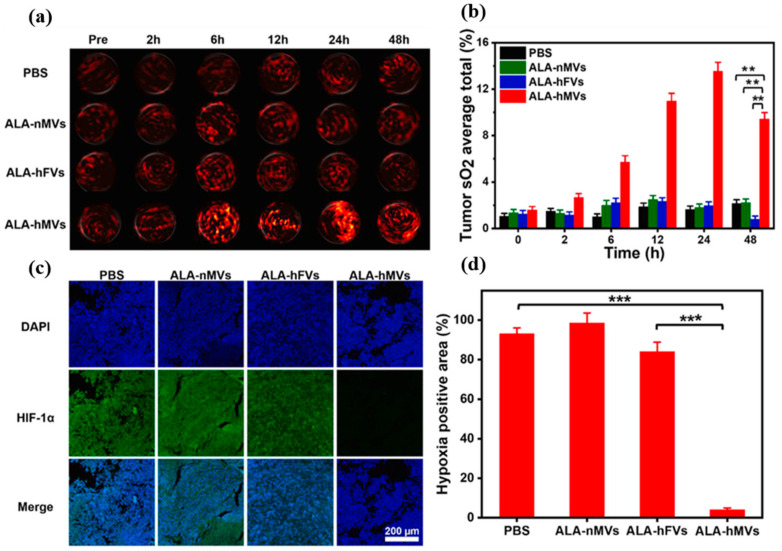
(**a**) Representative PA images of B16 tumors on mice showing signals of oxygenated hemoglobin (λ = 850 nm) before/after i.v. injection of various formulations. (**b**) The corresponding quantification of the tumor vascular saturated O_2_ levels (sO_2_) calculated from (**a**). (**c**) Immunofluorescence staining of tumor sections showing the expression of HIF-1α after i.v. injection of various formulations (**d**) Quantitative analysis of HIF-1α positive areas for each group in (**c**) by using the ImageJ software (*n* = 5). (*** *p* < 0.001, ** *p* < 0.01). Reproduced with permission [108]. Copyright 2021, Elsevier B.V.

Wu et al., investigated modulation in tumor oxygenation after i.v. administration of ICG, CAT loaded dendritic MSN. Changes in tumor sO_2_ in the four T1 tumors were evaluated by a Vevo-LAZR PAI system by measuring oxy-Hb and deoxy-Hb before and after injection. The results showed that sO_2_ signals in the PA image appeared at 2 h and became strong at 6 h, and signal intensity declined and became weak at 24 h. The quantitative analysis revealed an increase in average sO_2_ total to a maximum of ≈27% at 6 h from ≈10% at 2 h, and it decreased to ≈8% at 24 h. The CAT-loaded NP group increased the average sO_2_ total by approximately 3.94 and 4.43 times compared to control groups. The data obtained from quantitative sO_2_ analysis by PAI guided the authors to initiate PDT treatment at 6 h post-injection to increase the therapeutic performance of PDT [107]. Like PDT, SDT efficacy depends on oxygen tension in the tumor. Chen at al. developed hypoxia-responsive nanovesicles (hMVs) to alleviate hypoxia for enhanced PDT (Figure 6). When administered i.v., hMVs accumulated in the tumor followed by disassembly to release manganese ferrite NP to catalyze H_2_O_2_ to O_2_. The tumor oxygen saturation changes were monitored by PAI. Tumor sO_2_ levels were evaluated using PAI by measuring oxy-Hb and deoxy-Hb. The tumor vascular sO_2_ increased from 1.6 ± 0.3% (pre-injection) to 13.6 ± 0.8% at 24 h post-injection of nanovesicles, which was several-fold higher than that of controls. This indicates efficient tumor oxygenation and hypoxia relief by the hMVs, which was also confirmed by immunofluorescence staining of HIF-1α in tumor sections after 24 h post-injection. From the tumor sO_2_ values obtained from the quantitative PA analysis, SDT was applied at 24 h post-injection of hMVs, which resulted in significant inhibition in tumor growth [108].

Liu et al., developed biosynthetic functional vesicles (BFV) covered with PD1 and TRAIL and loaded with CAT to boost systemic antitumor immunity. After local injection of BFV, PAI was used to determine tumor sO_2_, and it was found that sO_2_ levels of BFV-treated tumor were comparable to CAT-only treated tumor. Furthermore, a reduction in hypoxia levels infiltrated cytotoxic T cells in the tumor. Overall, the immuno-modulating ability and robust antitumor immunity of BFVs facilitated a significant regression of tumor growth, prevention of abscopal tumors, and excellent inhibition of lung metastasis [109]. Liu et al., developed CAT@liposomes for oxygen-enriched radioimmunotherapy with CTLA4 blockade. To realize this, liposomes were individually encapsulated with CAT and H_2_O_2_, respectively. In vivo, CAT liposomes were i.v. administered first to consume H_2_O_2_ in TME to generate oxygen. After 4 h, H_2_O_2_ liposomes were injected, which amplified the tumor oxygenation confirmed by PAI analysis. The well-oxygenated tumors not only improved RT efficacy but also transformed cold immune suppressive-type TME to hot immune responsive type. When combined with immune checkpoint blockade, robust antitumor immune response was induced to destroy tumors [110]. 

Liu et al., developed an in situ gelation system by mixing immune adjuvant NP and PS-modified CAT together with PEG-diacrylate to induce immune responses after PDT. The mixed precursor solutions were locally injected followed by irradiation to induce gelation. The tumor-resident hybrid gel changed the tumor sO_2_ by decomposing H_2_O_2_ to O_2_. The changes were quantitatively measured using PAI. Tumors on mice treated with precursor materials without irradiation showed increased sO_2_ levels in the first 2 h. However, sO_2_ levels decreased at later time points due to rapid tumor clearance. When they irradiated the tumor with precursor materials, in situ gelation occurred, and their sO_2_ levels exhibited no significant increase in the first 2 h due to oxygen consumption by PDT. After 2 h, due to long-term tumor retention, tumors in this group showed greatly increased levels of sO_2_ even 48 h post-injection. In order to confirm tumor sO_2_ analysis by PAI, immunofluorescence staining of hypoxia was performed, which correlated with PAI results. In addition, multi-round PDT combined with a-CTLA4 inhibited metastasis offered long-term immune memory protection from tumor rechallenge [111].

## 5. Conclusions and Future Perspectives

Attributes of tumor hypoxia pose a significant risk and limitations to anticancer therapies, such as PDT, RT, and SDT. Reduced oxygen tensions in tumor hypoxia not only impair the production of ROS to kill cancer cells but also exacerbate the condition by consuming oxygen. As a consequence, it is quite evident that the detection and quantitative assessment of oxygen levels in the tumor are crucial. Specifically, it will assist to classify patients based on their hypoxic tumor status and guide optimal decision making in the therapy management of patients. Vast advances in interdisciplinary sciences, such as materials, physics and chemistry, have resulted in the development of sophisticated nanomaterials to increase tumor oxygenation levels by either releasing or generating oxygen. In this review, we summarized the following: (1) the key nanomaterial-based strategies to increase tumor oxygenation, which include (a) oxygen-releasing strategies, in which nanomaterials act as a host to oxygenphilic materials, such as PFCs and Hb, and (b) oxygen is generated by in situ reactions of CAT or CAT-like nanomaterials with endogenous H_2_O_2_, and (2) non-invasive PAI to monitor tumor hypoxia and quantify nanomaterials-mediated increase in tumor oxygenation in a real-time manner. 

Although the results are encouraging, four main concerns must be addressed prior to transition into clinics: (1) the feasibility of large-scale industrial production of these functional nanomaterials with quality control; (2) proper understanding of the in vivo fate of nanomaterials, such as their biodistribution and excretion; (3) since the premature release of oxygen can cause toxicity to normal tissues, controlled release of oxygen from nanomaterials is desired; and (4) due to dissipation of photons in the tissues, the penetration depth of PAI is currently restricted to ≈10 cm. Due to rapid advances in nanotechnology and PAI, we believe that the above challenges could be fully addressed to effectively detect, monitor, and overcome tumor hypoxia.

## Figures and Tables

**Table 1:** Summary of NP-mediated tumor sO_2_ increase quantified by PAI.

Design	Treatment Modality	Tumor sO_2_ Quantification by PAI	Ref
PFC-decorated tantalum oxide NP	RT	≈37% increase in tumor sO_2_ post i.t. injection	[70]
PFH-incorporated theranostic nanoemulsion	PDT	≈25% increase in tumor sO_2_ post i.v. injection	[72]
Hb-incorporated multifunctional nanocomplex	2 photon PDT	Strong PA signal from oxy-Hb were observed 6 h post-i.v. injection and continued to increase with time	[83]
HA-porous hollow Prussian blue NP	Tumor starvation therapy	≈35% increase in tumor sO_2_ 2 h post i.t. injection	[102]
Benzoporphyrin derivative as PS	PDT	Treatment responders exhibited ≈95% and ≈85% decrease in sO_2_ at 6- and 24-h post-PDT	[105]
Liposome-loaded DOX (HaT-DOX)	Chemotherapy	Treatment responders exhibited on average a 22% drop in sO_2_ 2 h post-chemotherapy.	[106]
Dendritic mesoporous organosilica NP-encapsulated ICG and CAT (ICG-CAT@MONs)	PAI/US guided PDT	≈27% increase in tumor sO_2_ 6 h post injection	[107]
Manganese ferrite NP embedded in hypoxia-responsive amphiphilic polymer membranes loaded with δ-aminolevulinic acid (ALA-hMVs)	SDT	The tumor vascular sO_2_ increased from 1.6 ± 0.3% (pre-injection) to 13.6 ± 0.8% at 24 h post-injection	[108]
Biosynthetic functional vesicles (BFVs) presenting PD1 and TRAIL on the surface, loading CAT in their inner core	Immunotherapy	Tumor sO_2_ levels in the BFVs/PD1-TRAIL-CAT or free CAT-treated tumors were significantly higher than PBS group	[109]
CAT@liposome	Radio combined Immunotherapy	Tumor sO_2_ levels increased to ≈32% at 24 h post injection of CAT@liposome combined H_2_O_2_@liposome	[110]
In situ gelation system containing PS-modified CAT together with PEG-double acrylate (PEGDA) as the polymeric matrix loading immune adjuvant NP	PDT combined immunotherapy	Tumor sO_2_ increased to ≈30% at 48 h post local injection	[111]
Self-delivery nanomedicine	PDT	Tumor sO_2_ increased to ≈45% at 6 h post i.v. injection	[112]
Tirapazamine-loaded metal–organic framework	Hypoxia activated therapy	Tumor sO_2_ decreased from ≈75% to ≈25% at 2 h post i.t. injection	[113]
Photoacoustic nanodroplets	PDT	Tumor sO_2_ increased to ≈9% post i.v. injection	[114]
Multifunctional theranostic NP	SDT and starvation therapy	Tumor sO_2_ increased to ≈18% at 24 h post i.v. injection	[115]
Biodegradable catalytic NP	Tumor catalytic therapy	Tumor sO_2_ increased to ≈40% post i.v. injection	[116]

## Data Availability

Data are contained within the article.

## Abbreviations

CDT	Chemodynamic therapy
CAT	Catalase
CTLA4	Cytotoxic T-lymphocyte-associated protein 4
DOX	Doxorubicin
FDC	Perfluorodecalyn
GOx	Glucoseoxidase
HIF-1α	Hypoxia-inducible factor-1α
Hb	Hemoglobin
HBOT	Hyperbaric oxygen therapy
HPV	Human papilloma virus
IDO	Indoleamine 2,3-dioxygenase
i.v.	Intravenous
i.t.	Intratumor
ICG	Indocyanine green
MRI	Magnetic resonance imaging
NP	Nanoparticles
NIR	Near infrared
pO_2_	Partial pressure of oxygen
PDT	Photodynamic therapy
PD-L1	Programmed death-ligand 1
PD-1	Programmed cell death protein 1
PS	Photosensitizer
PFC	Perfluorocarbon
PFOB	Perfluorooctyl bromide
PFH	Perfluorohexane
PFTA	Perfluorotributylamine
RT	Radiotherapy
ROS	Reactive oxygen species
sO_2_	Oxygen saturation
SDT	Sonodynamic therapy
SBH-PACT	Single breath hold-photoacoustic computed tomography
US	Ultrasound
